# Current Surgical Treatment for Neurogenic Lower Urinary Tract Dysfunction in Patients with Chronic Spinal Cord Injury

**DOI:** 10.3390/jcm12041400

**Published:** 2023-02-10

**Authors:** Yu-Hua Fan, Yuan-Chi Shen, Chih-Chen Hsu, Po-Ming Chow, Po-Chih Chang, Yu-Hua Lin, Shang-Jen Chang, Yuan-Hong Jiang, Chun-Hou Liao, Chung-Cheng Wang, Chun-Te Wu, Hann-Chorng Kuo

**Affiliations:** 1 Department of Urology, Taipei Veterans General Hospital, Taipei 11217, Taiwan; 2 Department of Urology, College of Medicine, National Yang Ming Chiao Tung University, Taipei 11217, Taiwan; 3 College of Medicine, Chang Gung University, Taoyuan 33302, Taiwan; 4 Department of Urology, Kaohsiung Chang Gung Memorial Hospital, Kaohsiung 83301, Taiwan; 5 Department of Urology, Taipei Hospital, Ministry of Health and Welfare, Taipei 24233, Taiwan; 6 Department of Urology, National Taiwan University Hospital and College of Medicine, Taipei 10002, Taiwan; 7 Department of Urology, Chang Gung Memorial Hospital at Linkou, Taoyuan 33305, Taiwan; 8 Department of Chemistry, Fu Jen Catholic University, New Taipei City 24205, Taiwan; 9 Department of Urology, Hualien Tzu Chi Hospital, Buddhist Tzu Chi Medical Foundation and Tzu Chi University, Hualien 97004, Taiwan; 10 Divisions of Urology, Department of Surgery, Cardinal Tien Hospital, New Taipei City 23148, Taiwan; 11 Department of Urology, En Chu Kong Hospital, New Taipei City 23702, Taiwan

**Keywords:** spinal cord injury, urologic surgery, neurogenic lower urinary tract dysfunction

## Abstract

This study aimed to present a comprehensive literature review of the efforts of a spinal cord injury workgroup in Taiwan regarding urologic surgery for neurogenic lower urinary tract dysfunction (NLUTD) in patients with chronic spinal cord injury (SCI). Surgical procedures should be viewed as a final option for managing patients with SCI who have persistent symptoms and complications that cannot be resolved by other means. Surgeries can be grouped according to their purpose: reducing bladder pressures, reducing urethra resistance, increasing urethra resistance, and urinary diversion. The choice of surgery depends on the type of LUTD based on urodynamic tests. Additionally, cognitive function, hand motility, comorbidities, efficacy of surgery, and related complications should be considered.

## 1. Introduction

The worldwide incidence of spinal cord injury (SCI) ranges from 12.1 to 57.8 per million [1]. The annual incidence of SCI was approximately 241 per million in Taiwan [2]. Neurogenic lower urinary tract dysfunction (NLUTD) occurs depending on the level and extent of the SCI. It is estimated that 70–84% of SCI patients have NLUTD [3]. The micturition center in the spinal cord is located at the S2–S4 levels [4]. Complete suprasacral injuries are typically associated with detrusor overactivity and detrusor sphincter dyssynergia. Conversely, complete sacral injuries results in hypocontractile or acontractile detrusor [5]. Nevertheless, incomplete SCI and coexistence of multiple injuries at different levels may cause unpredictable mixed NLUTD [6]. Urodynamic testing involves evaluating the function and dynamics of the lower urinary tract to assess the performance of the detrusor muscle and the bladder outlet. Urodynamic evaluation is paramount to correctly identify the underlying LUTD and to optimize long-term management.

There are three main types of NLUTD associated with SCI: (1) failure to store, (2) failure to empty, and (3) a combination of the two [7]. If the NLUTD is not treated properly, urological complications such as urinary tract infection (UTI), urolithiasis, urinary incontinence and upper urinary tract deterioration may develop consequently [8]. Chen et al. evaluated urological complications in different levels of SCI and reported that severe urinary incontinence occurred more often in patients with cervical and thoracic SCI, and urolithiasis was more frequent in patients with sacral SCI. Nevertheless, the frequency of symptomatic UTI and hydronephrosis was similar among different levels of SCI [9].

The main objective of treating NLUTD is to preserve the upper urinary tract by implementing a treatment plan that focuses on keeping the bladder pressure low, which helps to store urine safely. The secondary objective is to achieve urinary continence, prevent urological complications and enhance the patient’s overall quality of life. These objectives can be met through an comprehensive management program that includes self-catheterization and medications. Surgery should only be considered as a final resort for patients with SCI who have persistent symptoms and complications that cannot be resolved through other means.

Surgeries can be grouped according to their purposes: Reducing intravesical pressures, reducing urethral resistance, increasing urethra resistance, and urinary diversion.

## 2. Reducing Intravesical Pressures

### 2.1. Augmentation Cystoplasty

Indications for augmentation cystoplasty (AC) are low bladder capacity, poorly compliant bladder, autonomic dysreflexia or refractory overactive bladder with high intravesical pressure. Inability to perform clean intermittent catheterization (CIC), inflammatory bowel disease, significant prior small bowel resection and abdominopelvic radiation, and renal insufficiency are contraindications of AC [10].

The segment of the bowel that is most commonly used, well-researched, and generally preferred for AC is the detubularized ileum [11,12,13,14]. Meanwhile, it is uncertain if ureteric reimplantation is necessary during the AC procedure for patients with vesicoureteral reflux (VUR). Studies have shown that VUR often improves or disappears after AC, indicating that ureteral reimplantation may not be necessary for resolving symptoms of UTI and maintaining kidney function [15].

Urodynamic parameters significantly improved after AC. Hoen et al. conducted a systematic review on bladder augmentation in patients with neurogenic bladder dysfunction and reported that the average pre- and post-operative bladder capacities were 169 mL and 559 mL, respectively [16]. Furthermore, an average preoperative detrusor pressure at a maximum capacity of 65 cmH2O decreased to a median post-operative detrusor pressure of 19 cmH2O at follow-up [16]. A total of 83% of patients no longer had VUR, and 10% of patients showed improvement in VUR [17]. Urinary continence can be achieved in 90% of patients [18,19,20,21]. Furthermore, symptomatic UTI was reduced and autonomic dysreflexia improved in most patients after AC [22].

However, after AC, 10% of patients had persistent urinary incontinence caused by intrinsic sphincteric deficiency, [23] 93.3% of the patients were at least partially dependent on catheterization, and 76% of patients were totally catheter-dependent [24]. Previous studies reported that 0–29% of patients require antimuscarinics treatment after AC [19,25,26,27]. Renal function did not appear to be significantly different after AC, and the risk of development of end-stage renal disease was rare [16,24]. Other long-term complications are summarized in Table 1 [12,28,29,30,31,32,33,34,35,36,37].

### 2.2. Bladder Auto-Augmentation

Detrusor myotomy or myectomy is a surgical procedure of myotomy to increase bladder capacity without opening the bladder mucosa. The operation results in an increase in the diverticulum-like outward expansion of the urothelial mucosa and the functional bladder capacity. The advantages of detrusor myotomy or myectomy are their low surgical burden and low rates of long-term adverse effects, which positively affect patient quality of life [38,39,40,41].

The best outcome of bladder auto-augmentation could be expected in patients with NLUTD with a fair bladder capacity but poor bladder compliance [21]. However, because the presence of secondary fibrosis and the healing process of the divided detrusor muscle might occur, auto-augmentation yielded limited long-term improvement in the maximum cystometric capacity, maximum detrusor pressure, and bladder compliance compared to AC [42]. Additionally, the effectiveness of auto-augmentation in the long-term is not as high as AC, with a repeat of the AC procedure being required for 15–45% of patients [21,43,44,45].

### 2.3. Sacral Anterior Root Stimulation, Posterior Sacral Root Rhizotomy (SARS)

The combination of sacral anterior root stimulation and posterior sacral root rhizotomy (SARS), also referred to as the “Brindley procedure,” has been developed by G.S. Brindley [46] and D. Sauerwein [47]. The procedure is only indicated in patients with complete SCI. This procedure consists of an intradural rhizotomy of the afferent sacral nerves to abolish detrusor overactivity combined with the intradural implantation of an anterior root stimulator which produces an effective contraction that can empty the bladder.

Posterior sacral root rhizotomy suppresses detrusor overactivity which results in a low-pressure bladder and reduces autonomic dysreflexia. Posterior sacral root rhizotomy can also be carried out with intermittent catheterization to empty the bladder without implantation of an anterior root stimulator [48].

SARS treatment reduced the number of patients in danger of upper urinary tract damage by over 75% [49]. Continence was achieved in 70–91% of these patients [49,50,51]. Persisting autonomic dysreflexia was reported in 0–40% of patients [49,50,51,52]. The annual UTI rate was significantly reduced by over 50% [49].

In a previous report, 39% of patients receiving SARS needed surgical revision [49]. The most frequent (28%) cause for revision surgery was a malfunction of the stimulator cables or the receiver plate. SARS may influence reflex defecation and sexual function. Additionally, SARS involves a laminectomy, which may result in vertebral column instability in the setting of SCI-induced osteoporosis [49].

### 2.4. Sacral Neuromodulation

The American Urological Association/Society of Urodynamics Female Pelvic Medicine and Urogenital Reconstruction Adult Urodynamics guidelines state that clinicians should not offer sacral neuromodulation to patients with SCI and NLUTD due to the extensive variation in bladder dysfunction in this group [53]. Previous studies have shown that sacral neuromodulation (SNM) may improve incontinence, urinary tract infections, and upper tract protection in patients with SCI. Nevertheless, there were diverse clinical scenarios, and further revisions and additional procedures were also necessary [54,55].

Complete SCI is generally considered as a contraindication for SNM. Sievert et al. investigated the effect of early bilateral SNM in patients with complete SCI during the spinal shock phase and reported that early SNM in the shock phase might prevent the development of detrusor overactivity [56]. The promising preliminary results for early SNM in complete SCI should be confirmed in future investigations.

## 3. Reducing Urethra Resistance

### 3.1. External Sphincterotomy

External sphincterotomy should only be given to men who are able to use a condom catheter. It is an effective method for resolving autonomic dysreflexia, hydronephrosis, and recurrent UTI and for reducing detrusor pressures, post-void residual urine, and VUR. = [57,58]. The preferred is the 12 o’clock sphincterotomy with cold knife or neodymium-doped yttrium aluminum garnet (Nd:YAG) laser, owing to its fewer complications, such as hemorrhage and erectile dysfunction [59,60]. The reoperation rate was up to 57% [61].

### 3.2. Urethral Stent

Urethral stent placement is a potentially reversible treatment that is as effective as external sphincterotomy with shorter operative times and lengths of hospital stay [62]. However, the long-term results of urethral stent placement were not as satisfactory as expected, and many complications might develop. The most frequently encountered stent complication was device migration (28%), followed by stenosis (15%), lithiasis (6%), and intraprosthetic calcification (2%). In total, 8.5% of patients required stent removal [63]. Therefore, permanent urethra stents have been removed from the market. Temporary urethra stents, such as silicone-coated urethra stents which prevent the entrapment of the stents in the urethra, would be advised [64].

### 3.3. Transurethral Incision of Bladder Neck

Transurethral incision of bladder neck (TUI-BN) is indicated in patients with bladder neck dyssynergia and not suggested for patients with detrusor hypertrophy, which leads to thickening of the bladder neck [65]. TUI-BN is successful in enabling spontaneous voiding, improving maximum flow rate, and lowering post-void residual volume in high-level SCI patients with intact detrusor contractility who experience spontaneous voiding by reflex or abdominal percussion. TUI-BN also leads to improvement in reducing bladder outlet resistance, reduction in the occurrence of autonomic dysreflexia episodes, and improvement in quality of life [66].

### 3.4. Transurethral Resection of Prostate

Transurethral resection of prostate (TURP) might be provided in male patients with incomplete SCI who are able to void voluntarily and presented with benign prostatic obstruction. In selected patients with SCI and intact detrusor contractility, TURP yielded post-void residual volumes of less than 200 mL in 76% of male patients with SCI [67].

## 4. Increasing Urethra Resistance

### 4.1. Artificial Urinary Sphincter

The use of an artificial urinary sphincter (AUS) is the most common treatment offered for patients with neurogenic stress urinary incontinence [68,69,70,71,72,73,74,75,76,77,78,79,80]. The cure rate was 22–100% [73,74,75,76,77,78,79]. Among the SCI patients, 32% of them underwent simultaneous bladder augmentation [69,70,71,73,76,77,78]. New-onset or persistent detrusor overactivity was observed in 8% of patients, and 6% of patients subsequently required bladder augmentation [69,72,75,78]. High surgical revision rates have been reported in over 50% of patients following AUS failure, mainly due to mechanical failure [68,69,70,71,72,73,74,75,76,77,78,79,80]. AUS cuff erosion rates have been reported as high as 41% and 26% in women and men, respectively [68,69,70,71,72,73,74,75,76,77,78,79,80]. AUS should be provided to males with normal cognitive abilities and adequate manual finger dexterity to operate the device [81].

### 4.2. Sling Surgery

#### 4.2.1. Autologous Sling

Autologous sling achieved complete continence in 83% and 74% of women and men, respectively [82,83,84,85,86,87,88]. Overall, 50% of women and 78% of men underwent simultaneous bladder augmentation [83,84,85,86,88,89,90,91,92]. Nevertheless, detrusor overactivity rather than lower urethra resistance might be the reason for combining AC with concomitant autologous sling to achieve continence [93]. A total of 91% of men regularly performed self-catheterization before surgery, and no issues in catheterizing were noticed following fascial sling placement [87,88,89,90,91,92]. Erosion, vesicovaginal fistula, and urethral stenosis were rare complications [84].

#### 4.2.2. Synthetic Tapes

Complete continence was achieved using synthetic tapes in 79% and 50% of women and men, respectively, after suburethral synthetic sling [82,89,91,92,94,95,96]. Approximately 7–35% of women with spontaneous voiding before surgery needed to perform self-catheterization following sling surgery with synthetic tapes [82,94]. Difficult catheterization following sling surgery was observed in 11% of men performing self-catheterization regularly before surgery [89,90]. Vaginal mesh exposure was observed in approximately 2% of women [82,96].

### 4.3. Adjustable Continence Therapy

Adjustable continence therapy (ACT) is a technique that involved the use of two silicone balloons that are connected to a titanium and silicone port through conduits. These balloons are positioned in the periurethral space at the bladder neck under fluoroscopic imaging with the goal of augmenting urethral resistance and stabilizing the bladder neck. The ports are placed under the skin of the labia majora/scrotum, and this allows for postoperative adjustments of the balloon if needed [97]. The complete continence rate was 12% [98,99,100,101]. Device reimplantation was required in 27% of patients due to migration (30%), balloon infection (19%), urethra exposure (18%), cutaneous exposure (18%), or perforation (16%) [98,101].

### 4.4. Bulking Agents

Collagen and Macroplastique^®^ have been utilized to manage neurogenic urinary incontinence in patients with SCI [102,103]. The cure rate has been 35% [102,103]. No significant adverse events have been reported [102,103].

## 5. Urinary Diversion

Urinary diversion is indicated in SCI patients who have urethral erosion with a damaged bladder outlet or are unable to perform indwelling or intermittent catheterization. Urinary diversion could improve independence, ease of bladder management and quality of life. Generally, continent methods of urinary reconstruction are preferred over incontinent techniques. Incontinent diversion is typically used in patients with compromised renal function, intrinsic sphincter deficiency, impaired cognitive ability, failed continent diversion, and inability to perform self-catheterization [104].

### 5.1. Continent Diversion

Continent diversions are classified based on their continence mechanism: Tunneled channel and nipple valve.

#### 5.1.1. Tunneled Channel

Continence can be attained by creating a tunnel through the detrusor muscle, usually in the appendix (Mitrofanoff appendicovesicostomy) [105] or reconfigured ileum (Yang-Monti) [106,107], and connecting the end to the urothelium. Other alternative structures for tunneled channel include the cecum and appendix monobloc, bladder, stomach, distal ureter, Meckel’s diverticulum, preputial penile, or clitoral skin [108,109,110,111,112,113]. The site of cutaneous anastomosis is chosen based on the patient’s ability and the position used for self-catheterization. Interposition of an abdominal wall skin flap in the distal end of the channel, such as in the V, VQZ, or VR flap, is preferable to minimize stenosis of the circular orifice scars [114,115].

Stomal complications have been reported to occur in 16–60% patients with an appendicovesicostomy and in 10–23% with a Yang-Monti procedure [116,117]. A total of 75–100% and 63–100% of patients with an appendicovesicostomy and a Yang–Monti procedure, respectively, reported complete continence [118].

#### 5.1.2. Nipple Valve

Nipple valves ensure continence by circumferential coaptation. The ileocecal valve, which functions as a natural nipple valve, is most commonly used. Additionally, a capacious cecal augment can be created with minimal bowel resection when AC is indicated. The technique of intussuscepted ileum, which was introduced by Kock and colleagues in 1982 as a method for creating a catheterizable diversion, is rarely used due to its complexity, lack of consistency, and poor outcomes [119,120].

### 5.2. Incontinent Diversion

#### 5.2.1. Ileal Conduit

The ileal conduit is the most frequently used form of incontinent urinary diversion. However, the risk of upper tract deterioration increases with longer periods of follow-up. Concomitant cystectomy to avoid pyocystitis may be advisable [121]. Stenosis of the uretero-ileal anastomosis have been reported in 2.9–8.8% of patients [121,122]. Febrile urinary tract infection and urolithiasis were reported in 3–8% and 3–31% of patients, respectively [123,124,125,126,127].

#### 5.2.2. Ileovesicostomy

Ileovesicostomy is a surgical procedure in which the upper end of a section of ileum is connected to the bladder, while the lower end is brought out to the abdominal wall as an incontinent stoma. The benefits of ileovesicostomy over ileal conduit include the prevention of uretero-intestinal stricture, elimination of the risk of pyocystitis if the bladder is retained, preseravtion of the natural upper tract anti-reflux mechanism, and preservation of sexual function [93]. However, stenosis of the stoma or the ileovesical anastomosis was reported in 8–38% of patients [128,129]. Urolithiasis was reported in 0–25% of patients [128,129]. A total of 33–75% of patients required subsequent procedures, mostly due to stoma stenosis and urinary stones [130].

#### 5.2.3. Vesicostomy

The cutaneous vesicostomy procedure is infrequently used in adult patients with SCI. It involves making a large opening in the bladder and sewing it directly to the skin as an incontinent stoma. Stoma stenosis was reported in 6–18% of patients [131]. Urolithiasis was reported in 6–20% of patients [131]. End stage renal disease developed in 6–18% of patients with a mean period of 6–7 years [131]. Vesicostomy is not a suitable method of management for adults with SCI [93].

#### 5.2.4. Cutaneous Ureterostomy

The cutaneous ureterostomy procedure involves connecting the ureters directly to the skin as a non-continent form of diversion. However, it has a high rate of stenosis and is infrequently used today, except as a temporary solution before definitive therapy [93]. UTI (mostly uncomplicated) and urolithiasis were reported in 6.6–10% and 10–15.5% of patients, respectively [132].

## 6. Discussion

**Figure 1:**
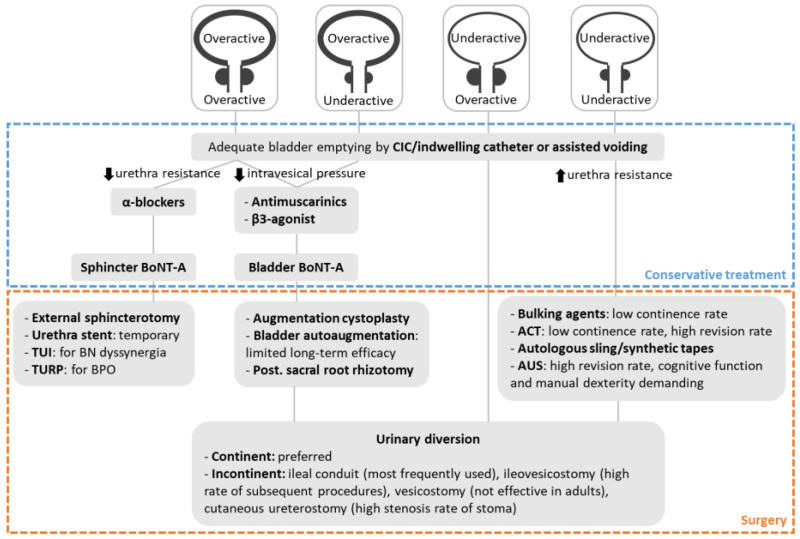
Algorithm of management of lower urinary tract dysfunction in patients with chronic spinal cord injury. BoNT-A, botulinum toxin A; TUI, transurethral incision; BN, bladder neck; TURP, Transurethral resection of prostate; BPO, benign prostatic obstruction; ACT, adjustable continence therapy; AUS, artificial urinary sphincter.

Urodynamic testing is mandatory to assess detrusor and bladder outlet function after SCI. Management of NLUTD in individuals with SCI should be based on urodynamic findings (Figure 1). According to the urodynamic results, the detrusor and urethra sphincter may be either overactive or underactive and display in four different combinations. Adequate bladder emptying is important to prevent UTI, high intravesical pressure and incontinence. Conservative bladder-emptying methods include CIC/indwelling catheterization, reflex voiding, or voiding by abdominal pressure. Reflex voiding and voiding by abdominal pressure should be avoided unless the intravesical pressure remains within 40 cmH2O [133]. Alpha-adrenergic antagonists and urethral sphincter botulinum toxin A injection may be effective in decreasing urethral resistance and improve voiding [134,135,136,137]. Surgeries such as external sphincterotomy may be considered to reduce urethral resistance in patients’ refractory to conservative treatments. Antimuscarinics and β3-adrenoceptor agonist can reduce detrusor overactivity and lower intravesical pressure [138,139]. The clinical efficacy of intravesical botulinum toxin A injection after failure of medication has been demonstrated [140]. Surgeries such as augmentation cystoplasty is indicated in patients’ refractory to conservative treatments. There are no available effective drugs for neurogenic sphincteric incompetence. Surgeries such as autologous sling are a mainstay in treatment of sphincteric incompetence. When no other therapy is successful, urinary diversion must be considered for the protection of the upper urinary tract and for the patient’s quality of life.

## 7. Conclusions

The choice of surgery depends on the type of lower urinary tract dysfunction. Surgeries can be grouped according to their purpose: reducing bladder pressures, reducing urethra resistance, and increasing urethra resistance. The cognitive function, hand motility, comorbidities, and efficacy of surgery should also be considered for making an informed decision on surgery type. Urinary diversion may be left as a last option for the protection of the upper urinary tract and for the patient’s quality of life.

## Figures and Tables

**Table 1:** Complications after augmentation cystoplasty.

Complication	Frequency (%)
Urinary stones	5–52
Perforation	<1–14
Bowel dysfunction	<10–55
Malignancy	<1.0–5.5
Metabolic abnormalities	<5
Symptomatic UTI	4–43

## Data Availability

Not applicable.
